# Association between uncooperativeness and the glucose metabolism of patients with chronic behavioral disorders after severe traumatic brain injury: a cross-sectional retrospective study

**DOI:** 10.1186/s13030-018-0125-0

**Published:** 2018-04-23

**Authors:** Tomohiro Yamaki, Kosuke Suzuki, Yusuke Sudo, Tomihisa Niitsu, Masahiko Okai, Nobuo Oka, Masaru Odaki

**Affiliations:** 1 Division of Neurosurgery, Chiba Ryogo Center, National Agency for Automotive Safety and Victims’ Aid, 3-30-1 Isobe, Mihama-ku, Chiba, 261-0012 Japan; 2 Division of PET imaging, Chiba Ryogo Center, National Agency for Automotive Safety and Victims’ Aid, Chiba, Japan; 3 Department of Psychiatry, Graduate School of Medicine, Chiba University, Chiba, Japan

**Keywords:** Behavioral disorder, 18F-FDG-PET, Traumatic brain injury

## Abstract

**Bakground:**

Patients with behavioral disorders following severe traumatic brain injury (sTBI) often have disorders of consciousness that make expressing their emotional distress difficult. However, no standard method for assessing the unsettled and unforeseen responses that are associated with behavioral disorders has yet to be established. Because the thalamus is known to play a role in maintaining consciousness and cognition, we used 18F-fluorodeoxyglucose positron emission tomography/computed tomography (18F-FDG-PET/CT) to examine the association between brain glucose metabolism in the thalamus and behavioral disorders.

**Methods:**

We retrospectively analyzed 70 consecutive patients with sTBI who had been involved in motor vehicle accidents. To assess behavioral disorders, we evaluated 18 symptoms using the Brief Psychiatric Rating Scale (BPRS): Emotional Withdrawal, Conceptual Disorganization, Tension, Mannerisms and Posturing, Motor Retardation, Uncooperativeness, Blunted Affect, Excitement, Somatic Concern, Anxiety, Feeling of Guilt, Grandiosity, Depressive Mood, Hostility, Suspiciousness, Hallucinatory Behavior, Unusual Thought Content, and Disorientation. First, we identified clinical characteristics of sTBI patients with behavioral disorders. Next, we retrospectively analyzed 18F-FDG-PET/CT data to assess how thalamic activity was related with abnormal behaviors.

**Results:**

Twenty-six patients possessed the minimum communicatory ability required for psychiatric interview. Among them, 15 patients (57.7%) were diagnosed with behavioral disorder, 14 of whom had reached a stable psychiatric state after about 426.6 days of treatment. Excitement (13 patients) and uncooperativeness (10 patients) were the most frequently observed symptoms. Available 18F-FDG-PET/CT data indicated that thalamic glucose metabolism was imbalanced and lateralized (*p* = 0.04) in 6 patients who exhibited uncooperativeness.

**Conclusions:**

Behavioral symptoms of excitement and uncooperativeness were common in patients with sTBI, although most symptoms improved as the chronic stage continued. Our data support the idea that imbalanced laterality of glucose metabolism in the thalamus might be related to behavioral disorders characterized by uncooperativeness.

**Trial registration:**

UMIN 000029531. Registered 27 March 2017, retrospectively registered.

**Electronic supplementary material:**

The online version of this article (10.1186/s13030-018-0125-0) contains supplementary material, which is available to authorized users.

## Background

In the chronic stage of severe traumatic brain injury (sTBI), patients can recover from disorders of consciousness including unresponsive wakefulness syndrome (vegetative state; patients awake from coma but remain unresponsive) and minimally consciousness state [1–4]. However, the chronic stage of neurorehabilitation is often accompanied by behavioral disorders, while the arousal sate and neurological status are typically unstable [1–8]. Most patients with sTBI have difficulty expressing their emotional distress because of disorders of consciousness, disrupted higher brain function, and verbal disturbance such as tracheostomy [9–13]. However, for patients who can communicate at some basic level, emotional explosions and abnormal behaviors are often observed as nonverbal responses over the clinical course following neurorehabilitation [9, 10, 14–16]. Consequently, therapists and caregivers are adversely affected by this unsettling patient behavior that is based on insufficient communication [1, 2, 8–10, 16].

Recently, the thalamus was shown to play a role in cognition, the maintenance of consciousness, and the modulation of arousal and alertness [17–19]. In this study, we searched for clinical features common to patients with sTBI who exhibit behavioral disorders to determine if abnormal thalamic activity is associated with behavioral disorders. We assessed thalamic activity as glucose metabolism, objectively measured by18F-fluorodeoxyglucose positron emission tomography/computed tomography (18F-FDG-PET/CT).

The Brief Psychiatric Rating Scale (BPRS) assesses a large number of behaviors including (1) Emotional Withdrawal, (2) Conceptual Disorganization, (3) Tension, (4) Mannerisms and Posturing, (5) Motor Retardation, (6) Uncooperativeness, (7) Blunted Affect, (8) Excitement, (9) Somatic Concern, (10) Anxiety, (11) Feelings of Guilt, (12) Grandiosity, (13) Depressive Mood, (14) Hostility, (15) Suspiciousness, (16) Hallucinatory Behavior, (17) Unusual Thought Content, and (18) Disorientation. Rating of these 18 symptoms presents a quantitative measure of the overall mental status and degree of abnormal behavior [20].

## Methods

### Study design

This was a retrospective, single institutional study of inpatients with sTBI from June 2013 to July 2017. Informed consent to use medical records and images were obtained from the patients’ legal representatives. The study was conducted after approval by the institutional ethics committee (2017–14). A website with additional information and an opt-out option was set up and patients’ legal representatives were informed (http://www.chiba-ryougo.jp/publics/index/194/).

### Patients

Seventy-three patients with sTBI were enrolled. All patients had a Glasgow Coma Scale (GCS) score of ≤8 at the time of their accident and severe verbal disturbance due to aphasia, mutism, tracheostomy, dysthymia, communication disorder, or voice disturbance. This study excluded patients with a history of psychiatric disorders.

### Clinical assessment

Clinical assessments were performed by neurosurgeons and psychiatrists. The Coma Recovery Scale-Revised (CRS-R) and BPRS-based classification were used to assess clinical manifestation [20, 21]. The original BPRS includes 18 items scored from 0 (absent) to 7 (extremely severe); however, rather than using the 8-point-scale, we only judged whether or not a patient exhibited each symptom, as has been described previously [20, 22–24].

We divided BPRS-based classification into a verbal communication domain and non-verbal communication categories. The non-verbal communication category included eight BPRS symptoms (Emotional Withdrawal, Conceptual Disorganization, Tension, Mannerisms and Posturing, Motor Retardation, Uncooperativeness, Blunted Affect, and Excitement) and the verbal category contained the other 10 symptoms (Somatic Concern, Anxiety, Feelings of Guilt, Grandiosity, Depressive Mood, Hostility, Suspiciousness, Hallucinatory Behavior, Unusual Thought Content, and Disorientation) When we could not definitively determine if a patient exhibited a particular symptom, the item was regarded as negative because of disorders of consciousness, disrupted higher brain function, or verbal disturbance that precluded patients from explicitly expressing the condition. Psychiatrists interviewed the patients once a week during hospitalization, and the BPRS-based assessment was conducted at the first psychiatric interview.

We determined the time between onset and remission of behavioral disorders using the date of the first psychiatrist intervention as the starting point and the date that the psychiatrist noted “stable” in the medical record as the end point. BPRS and CRS-R scores were used at the same time on medical records.

### 18F-FDG-pet/CT

The exclusion criteria for 18F-FDG-PET/CT imaging were as follows: symptomatic status epilepticus, medical instability, uncooperative behavior or poor glycemic control. Patients were intravenously injected with 350 MBq of 18F-FDG following a period of at least 7 h of fasting. Image acquisition (Discovery® ST-E PET/CT, GE Healthcare, Tokyo, Japan) was then performed after 60 min.

The primary objective was to measure the maximum standardized uptake value (SUVmax) of the whole brain, each hemisphere, and the thalamus using three-dimensional volumes of interest (VOIs) based on commercial software (syngo.via®, Siemens, Tokyo, Japan). VOIs were set at 50% of the peak SUV value (Additional file 1: Figure S1).

### Statistical analyses

Statistical analyses were performed using JMP10® statistical software (IBM Japan, Tokyo, Japan). Statistical significance was calculated using Fisher’s exact tests, Chi-squared tests, and Student t tests. We did not correct for multiple comparison because we a priori focused on thalamic function. Data are presented as the mean ± standard deviation (SD). Results were considered statistically significant if the *p*-value was less than 0.05.

## Result

### Clinical symptoms

**Fig. 1:**
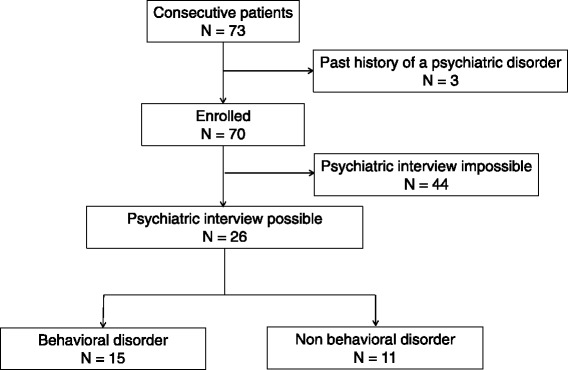
Study design and analysis profile. Psychiatric interview and behavioral disorders

Three patients with a history of a psychiatric disorder were excluded (Fig. 1). The mean time between the accident and admission to the hospital was 623 days. Clinical manifestations included severe disability in 27 patients (CRS-R score 19.6), minimally consciousness state in 13 (CRS-R score 14.1), and unresponsive wakefulness syndrome in 30 (CRS-R score 4.5; *p* < 0.0001).

Of the 70 enrolled patients, 44 presented with poor wakefulness, consciousness, or linguistic ability, which made psychiatric assessment impossible. Clinical manifestations in the remaining 26 patients were less severe, and they possessed at least the minimum level of communicative ability that allowed psychiatric assessment (Additional file 2: Table S1). Of these 26 patients, 15 patients (57.7%) were diagnosed with behavioral disorders (BD group) while the remaining 11 (42.3%) were not (Non-BD group). Neither the total CRS-R nor the subscale scores significantly differed between the BD group and the Non-BD group (Table 1).

**Table 1:** Comparison of severe traumatic brain injury patients with and without behavioral disorders

Demographic and clinical data	sTBI with behavioral disorder (*n* = 15)	sTBI without behavioral disorder (*n* = 11)	*P*
Age (year), mean (SD)	46.7 (12.3)	49.5 (21.9)	0.67
Male, n (%)	12 (80)	8 (72.7)	1
The sum of CRS-R at admission, mean (SD)	20.3 (3.0)	18.8 (2.7)	0.21
Auditory function score, mean (SD)	3.4 (0.1)	3.3 (0.9)	0.61
Visual function score, mean (SD)	4.5 (1.3)	4.3 (1.5)	0.72
Motor function score, mean (SD)	5.6 (0.6)	5.3 (0.9)	0.29
Oromotor/verbal function score, mean (SD)	2.3 (0.8)	1.8 (0.9)	0.14
Communication score, mean (SD)	1.4 (0.6)	1.2 (0.6)	0.38
Arousal score, mean (SD)	3.0 (0)	3.0 (0)	ND
Symptoms of behavioural disorder			
Somatic concern, n (%)	3 (20)	0 (0)	0.24
Anxiety, n (%)	2 (13.3)	0 (0)	0.49
Emotional withdrawal, n (%)	2 (13.3)	0 (0)	0.49
Conceptual disorganization, n (%)	1 (6.7)	0 (0)	1.00
Feelings of guilt, n (%)	0 (0)	1 (9.1)	0.42
Tension, n (%)	0 (0)	0 (0)	ND
Mannerisms and posturing, n (%)	5 (33.3)	0 (0)	0.05
Grandiosity, n (%)	0 (0)	0 (0)	ND
Depressive mood, n (%)	4 (26.7)	0 (0)	0.11
Hostility, n (%)	5 (33.3)	0 (0)	0.05
Suspiciousness, n (%)	1 (6.7)	0 (0)	1.00
Hallucinatory behaviour, n (%)	3 (20)	0 (0)	0.24
Motor retardation, n (%)	3 (20)	0 (0)	0.24
Uncooperativeness, n (%)	10 (66.7)	1 (9.1)	0.005*
Unusual thought content, n (%)	2 (13.3)	0 (0)	0.49
Blunted affect, n (%)	4 (26.7)	0 (0)	0.11
Excitement, n (%)	13 (86.7)	1 (9.1)	0.002*
Disorientation, n (%)	5 (33.3)	0 (0)	0.05

*P < 0.05; *CRS-R*, Coma Recovery Scale-Revised; *ND*, not detected; *SD*, standard deviation; *sTBI*, severe traumatic brain injury.

**Fig. 2:**
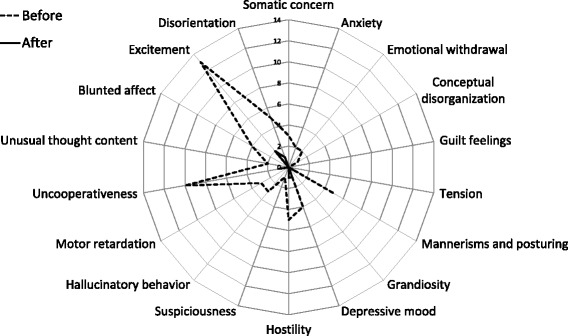
Total numbers of the abnormal behaviors obtained from 26 patients with severe traumatic brain injury before and after psychiatric intervention. Behaviors are based on Brief Psychiatric Rating Scale classification system

In the BD group, Excitement (86.7%) and Uncooperativeness (60%) were well observed as representative symptoms. We found no significant differences in age, sex, or auditory, visual, motor, oromotor/verbal, or arousal-related neurological functions between the BD and Non-BD groups. As shown in Fig. 2 and Tables 2, 14 members (93.3%) of the BD group reached a psychiatrically stable state after treatment, with the mean latency for psychiatric stability (starting from the beginning of psychiatric treatment) 426.6 ± 342.3 days. Psychiatric treatments for BD patients with psychiatric stability included sodium valproate (*n* = 7), quetiapine fumarate (n = 7), and aripiprazole (*n* = 4).

**Table 2:** Demographic and clinical data of severe traumatic brain injury patients with behavioral disorders

Demographic and clinical data	sTBI with behavioral disorder (*n* = 15)
Representative symptom	
Excitement, n (%)	13 (86.7)
Uncooperativeness , n (%)	9 (60)
Psychiatric stable state, n (%)	14 (93.3)
The duration until psychiatric stability, mean (SD)	426.6 (342.3)
The kinds of effective psychiatric drugs	
Sodium valproate increase or start, n (%)	7 (50)
Quetiapine fumarate increase of start, n (%)	7 (50)
Aripiprazole start, n (%)	4 (28.6)

*SD*, standard deviation; *sTBI*, severe traumatic brain injury.

### 18F-FDG-pet/CT

To find the thalamic activity patterns that were characteristics of behavioral disorders, we measured the SUVmax of the thalamus with 18F-FDG-PET/CT. Scanning image data were available for 14 patients (Table 3, Additional file 3: Table S2, Additional file 4: Figure S2). To take the side of injury into account, we calculated the laterality ratio of SUVmax for the left and right regions as LR ratio = SUVmax_left_/ SUVmax_right_.

**Table 3:** Comparison of severe traumatic brain injury (sTBI) patients with and without symptoms of uncooperativeness in right hemisphere, left hemisphere, right thalamus, left thalamus, the laterality ratio of SUVmax for the left and right regions as LR ratio = SUVmax_left_/ SUVmax_right_ of hemisphere, and thalamus by glucose uptake based on 18F-fluorodeoxyglucose positron emission tomography/computed tomography

	SUVmax ± SD (95% CI)		
Target of VOIs	sTBI with Uncooperativeness *n* = 6	sTBI without Uncooperativeness *n* = 8	*P*
Whole brain	11.94 ± 3.4 (8.37– - 15.51)	11.53 ± 3.3 (8.77– - 14.28 )	0.82
Right hemisphere	12.17 ± 3.83 (8.15– - 16.19 )	12.24 ± 4.6 (8.39– - 16.09)	0.98
Left hemisphere	10.89 ± 2.98 (7.76– - 14 )	11.33 ± 4.58 (7.5– - 15.16)	0.84
LR ratio of hemisphere	0.91 ± 0.11 (0.8– - 1.02)	0.93 ± 0.09 (0.85– - 1 )	0.73
Right thalamus	8.23 ± 2.57 (5.53– - 10.93)	6.76 ± 1.73 (5.32– - 8.21 )	0.23
Left thalamus	6.04 ± 2.28 (3.64– - 8.44)	7.25 ± 2.59 (5.09– - 9.41 )	0.38
LR ratio of thalamus	0.75 ± 0.11 (0.51– - 0.99)	1.09 ± 0.1 (0.88– - 1.3)	0.04*

*P < 0.05; *CI*, confidence interval; *LR ratio*, SUVmax_left_ / SUVmax_right_; *SD*, standard deviation; *sTBI*, severe traumatic brain injury; *SUVmax*, maximum standardized uptake value; *VOI*, volume of interest

The sample size was large enough for statistical analyses of SUVmax to be performed for the BPRS-classification categories of Excitement and Uncooperativeness. No significant differences were found between patients who did or did not exhibit Excitement (Additional file 5: Table S3). However, those who exhibited Uncooperative behavior displayed imbalanced laterality of glucose metabolism in thalamus compared with those who did not (*p* = 0.04).

## Discussion

Here, we show that Excitement and Uncooperativeness are the primary abnormal behaviors in chronic sTBI. 18F-FDG-PET/CT imaging revealed imbalanced laterality of thalamic glucose metabolism in those who were Uncooperative, pointing to a role for the thalamus in pathologically Uncooperative behavior.

To date, behavioral disorders in sTBI and mild TBI have been grouped together when discussed [1, 2, 8]. Recently, TBI pathology appears to differ depending on the severity of the brain injury [1, 2, 8]. Indeed, depression and mood disorder are commonly observed in patients with mild TBI [25]. However, unsettling and unforeseen reactions to therapists and caregivers is a common occurrence during neurorehabilitation for patients with sTBI [6, 7, 9, 10, 12, 26–29]. Therefore, neurological assessment and psychiatric assessment have not been able to segregate patients for managing treatment.

Although the CRS-R scale is widely used to assess clinical manifestations in patients with sTBI, it is not sufficient for appropriate mental and behavioral assessment [3, 21, 23]. This is because factors such as tracheotomy, the impairment of verbal function, aphasia and fluctuation of awareness, and disrupted higher brain function disturb self-expression in these patients [9–12, 26–29]. Our data show that patients with sTBI have a broad variety of symptoms. In the future, we must consider adding nonverbal assessment such as the visual analogue scale and the face-pain scale for detecting posttraumatic agitation, aggression, irritability, and sleep disorders, because these common symptoms are not included in the BPRS-based classification [10, 16, 28–31].

To assess their emotional distress, we chose a nonverbal, objective image analysis of the thalamus. 18F-FDG-PET/CT has been established as a useful technique [32–35], with one study showing that cortico-thalamo-cortical glucose metabolism was related to brain dysfunction in patients with sTBI [36]. Additionally, the thalamus is known to have a major role in cognitive function, maintenance of wakeful states, and neuroplasticity [17–19, 37, 38]. Our data showed imbalanced glucose metabolism in the thalamus of the patients who displayed Uncooperativeness. Glucose metabolism in the left thalamus was lower than in the right for Uncooperative patients. This might be related to language function, which is typically left-dominant [39]. Further, the imbalanced metabolism in the thalamus might influence Papez’ or Yakovlev’ limbic connection related to emotional control, resulting in uncooperativeness behavior following neurorehabilitation [38–43]. Importantly, most patients’ behavioral disorders improved to some extent after psychiatric treatment during the clinical course. Based on our data, sodium valproate and quetiapine fumarate have the potential to be effective therapeutic drugs for these behavioral disorders. These drugs might have an influence on FDG uptake because some psychotropic agents can change glucose metabolism in some brain areas [44–46]. We will soon begin designing a prospective trial using 18F-FDG-PET/CT to determine if these drugs can improve the imbalance in brain-glucose metabolism.

A major limitation of the present study is the probability of false positive findings due to the small sample size. A small sample was unavoidable because patients with behavioral disorders did not always cooperate with medical treatment, continuous rehabilitation, or image acquisition. An appropriate psychiatric approach that includes drugs and assessment will need more detailed information and require further investigation in a large sample.

## Conclusion

We found that Excitement and Uncooperativeness were the most commonly observed abnormal behaviors in patients with sTBI over the course of rehabilitation and care. Most abnormal behaviors improved over time. 18F-FDG-PET/CT images revealed imbalanced laterality of glucose metabolism in the thalamus of pathologically Uncooperative patients with sTBI.

## Additional files

Additional file 1: **Figure S1.** Representative images of a three-dimensional volume of interest measurement (a) and color mapped image (b) of glucose metabolism measured via 18F-fluorodeoxyglucose positron emission tomography/computed tomography. (PPTX 380 kb)

Additional file 2: **Table S1.** Comparison between the patients who were evaluated for psychiatric assessment (the evaluable group) and those who could not be assessed (the unevaluable group). (DOCX 18 kb)

Additional file 3: **Table S2.** Clinical information for 14 patients who had severe traumatic brain injury and provided 18F-fluorodeoxyglucose positron emission tomography/computed tomography images. Patients are separated based on whether or not they were diagnosed with a behavioral disorder. (XLSX 10 kb)

Additional file 4: **Figure S2.** All available 18F-fluorodeoxyglucose positron emission tomography/computed tomography images for the 14 patients who had severe traumatic brain injury with or without a behavioral disorder. (PPTX 885 kb)

Additional file 5: **Table S3.** Comparison of glucose uptake in patients who had severe traumatic brain injury, with or without non-verbal symptoms defined by the Brief Psychiatric Rating Scale classification system (Anxiety, Feelings of Guilt, Mannerisms and Posturing, Depressive Mood, Hostility, Hallucinatory Behavior, Motor Retardation, Blunted Affect, Excitement). Data are presented for the right hemisphere, left hemisphere, right thalamus, left thalamus, hemispheric LR ratio, and thalamic LR ratio. Glucose uptake is based on 18F-fluorodeoxyglucose positron emission tomography/computed tomography.*P < 0.05, *CI*, confidence interval; *LR ratio*, laterality ratio of SUVmax for the left and right regions; *SD*, standard deviation; *sTBI*, severe traumatic brain injury; *SUVmax*, maximum standardized uptake value; *VOI*, volume of interest. (XLSX 16 kb)

## Abbreviations

18F-FDG-PET/CT: 18F-fluorodeoxyglucose positron emission tomography/computed tomography
BD: Behavioral disorder
BPRS: Brief Psychiatric Rating Scale
CRS-R: Coma Recovery Scale-Revised
GCS: Glasgow Coma Scale
LR ratio: Laterality ratio of SUVmax for the left and right regions
SD: Standard deviation
sTBI: Severe traumatic brain injury
SUVmax: Maximum standardized uptake value
VOIs: Volumes of interest
