# Supplementary figures and images for: Association between uncooperativeness and the glucose metabolism of patients with chronic behavioral disorders after severe traumatic brain injury: a cross-sectional retrospective study
Source: Biopsychosoc Med. 2018 Apr 23;12:6. doi: 10.1186/s13030-018-0125-0 (PMC5914015; doi:10.1186/s13030-018-0125-0)

## Slide 1
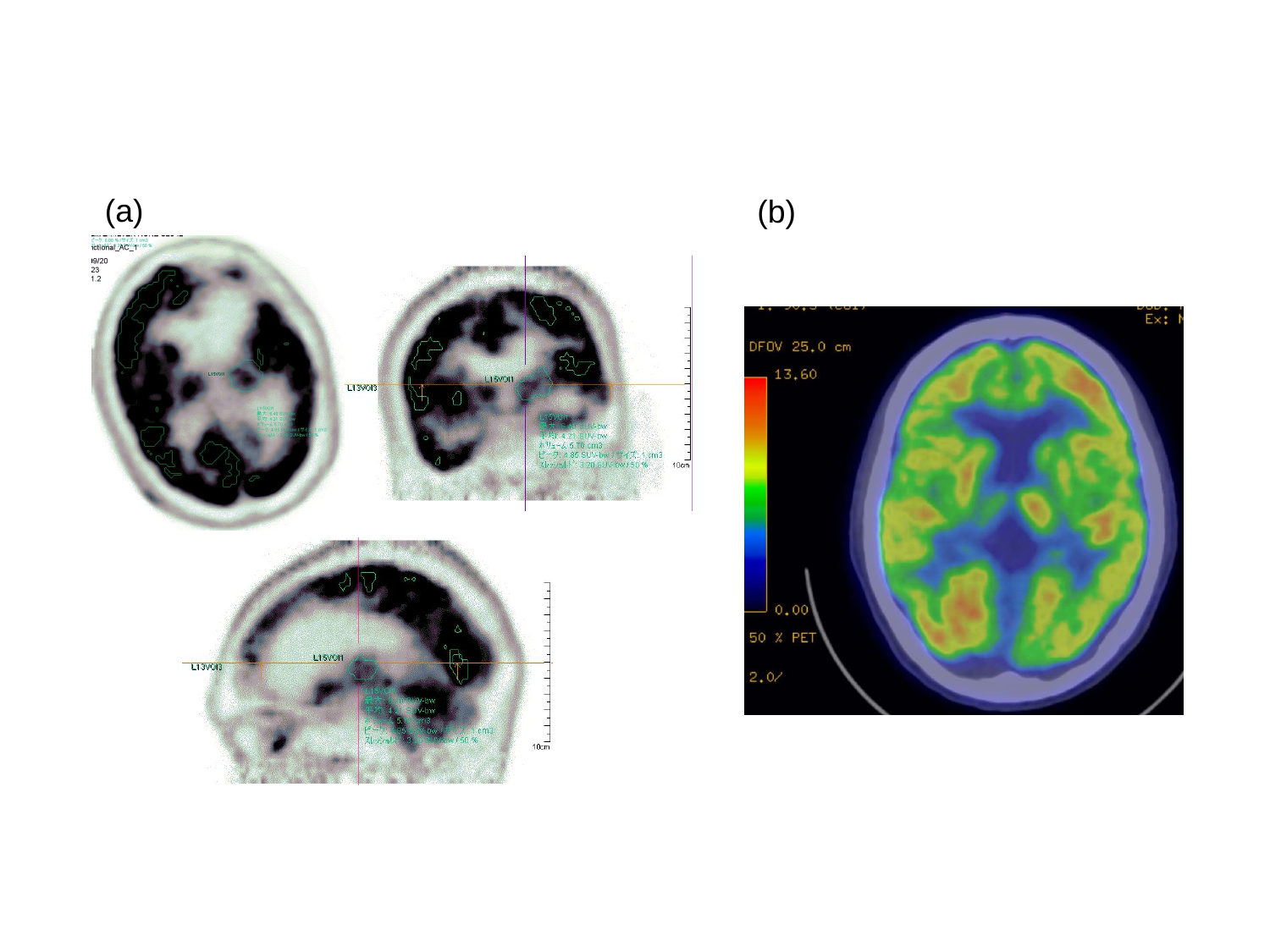

(a)
(b)

Supplement: Supplementary file 1 — Figure S1. Representative images of a three-dimensional volume of interest measurement (a) and color mapped image (b) of glucose metabolism measured via 18F-fluorodeoxyglucose positron emission tomography/computed tomography. (PPTX 380 kb) [file 13030_2018_125_MOESM1_ESM.pptx]

## Slide 1
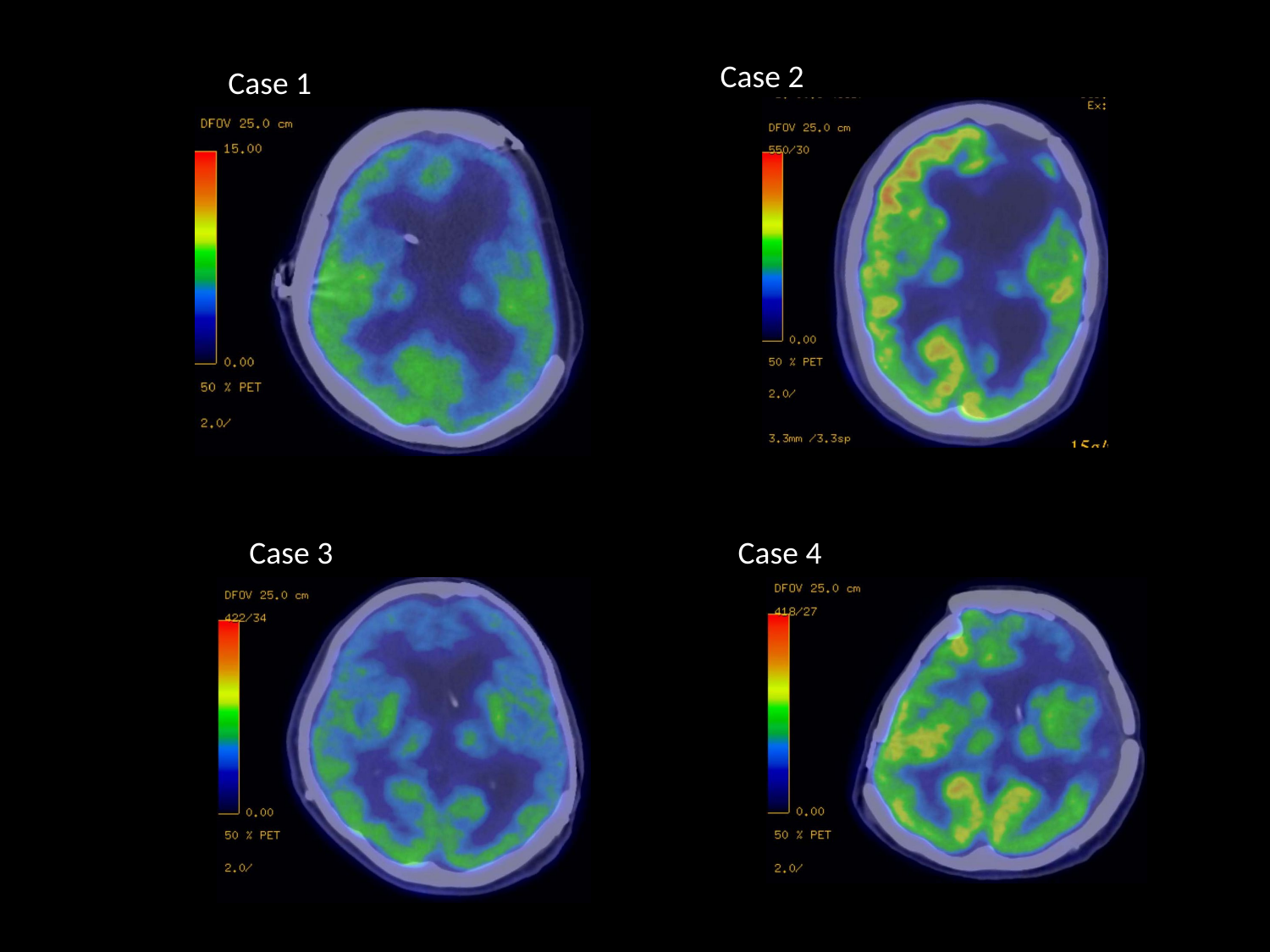

Case 2
Case 1
Case 3
Case 4

## Slide 2
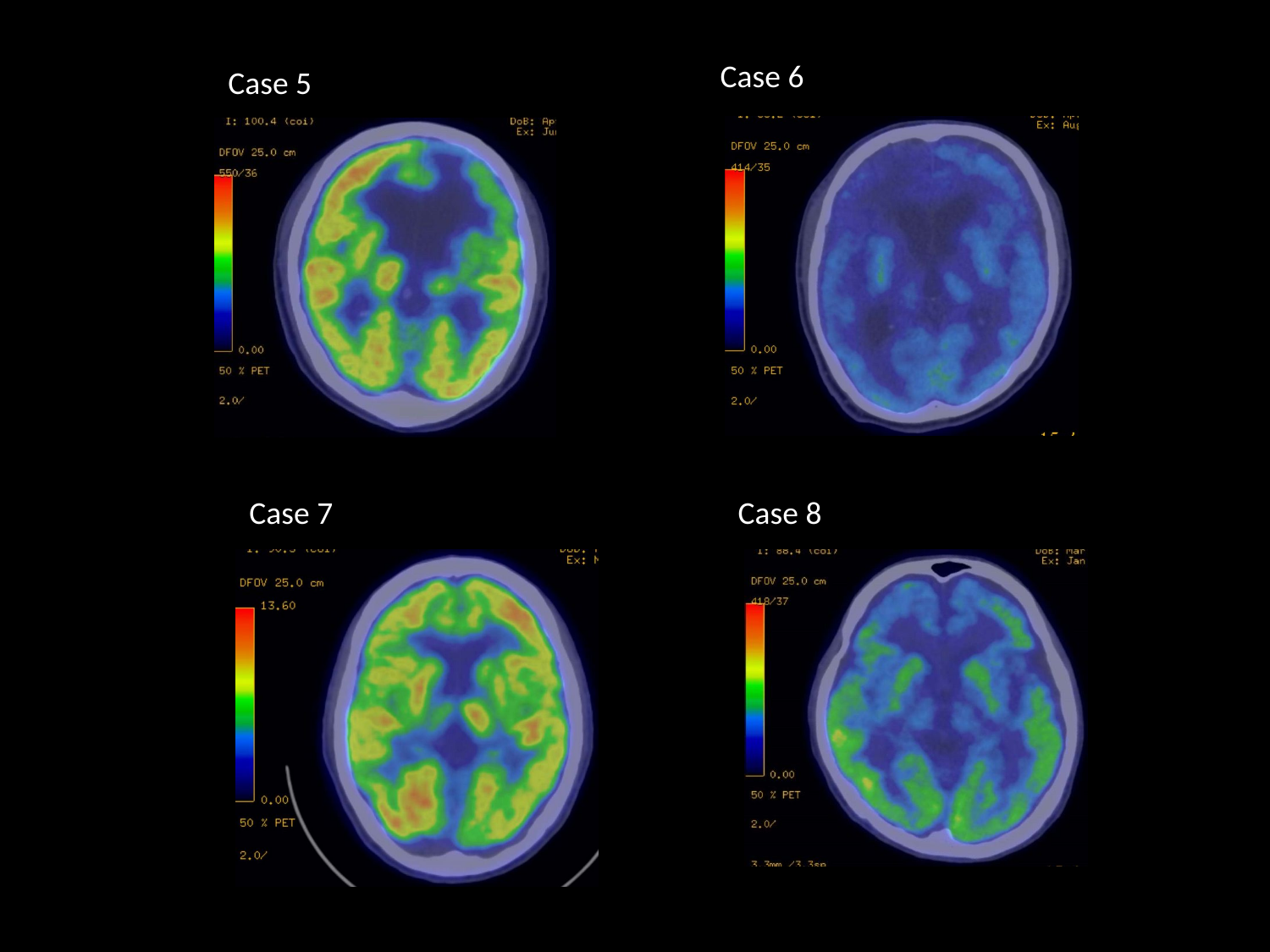

Case 6
Case 5
Case 7
Case 8

## Slide 3
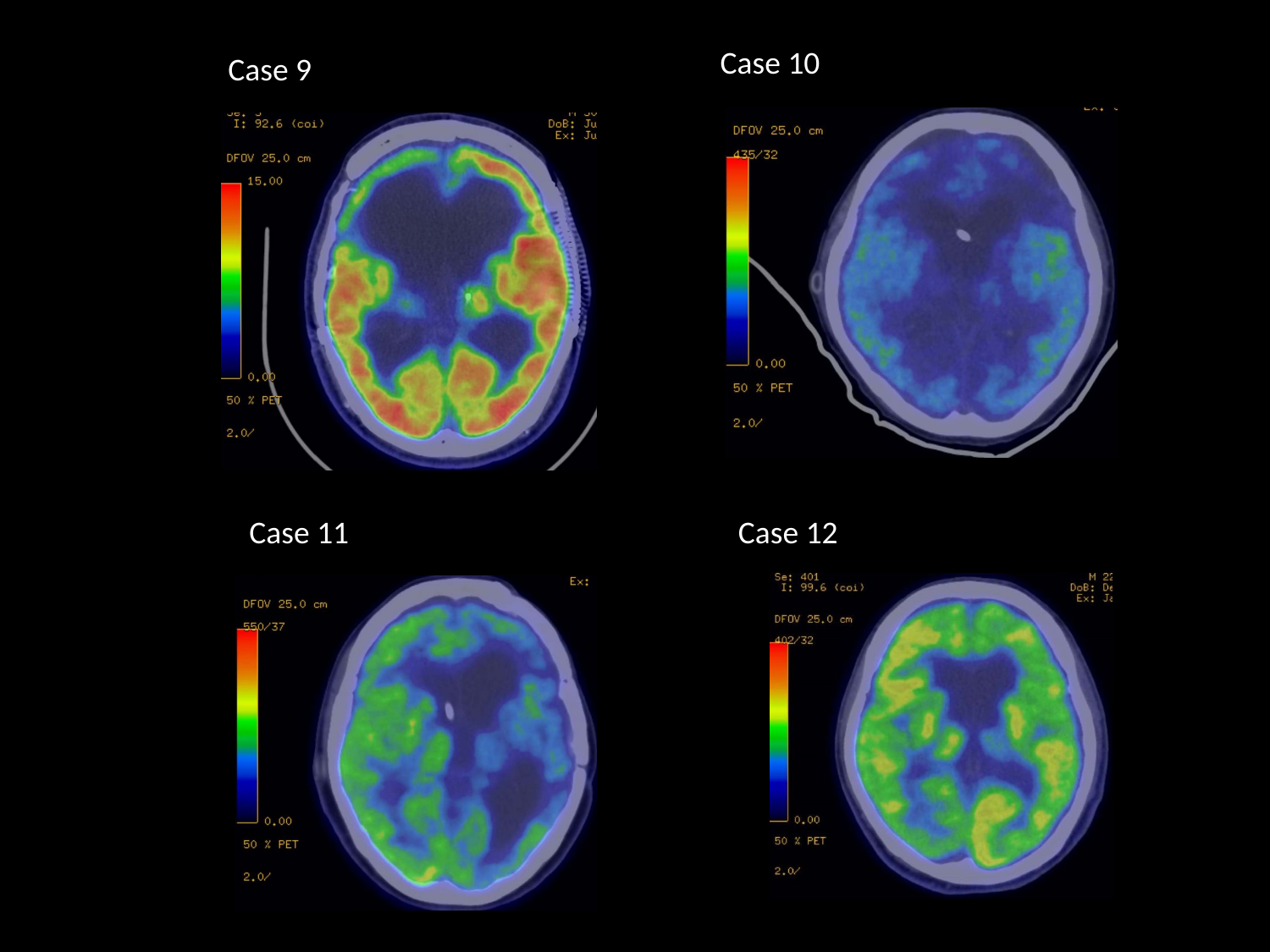

Case 10
Case 9
Case 11
Case 12

## Slide 4
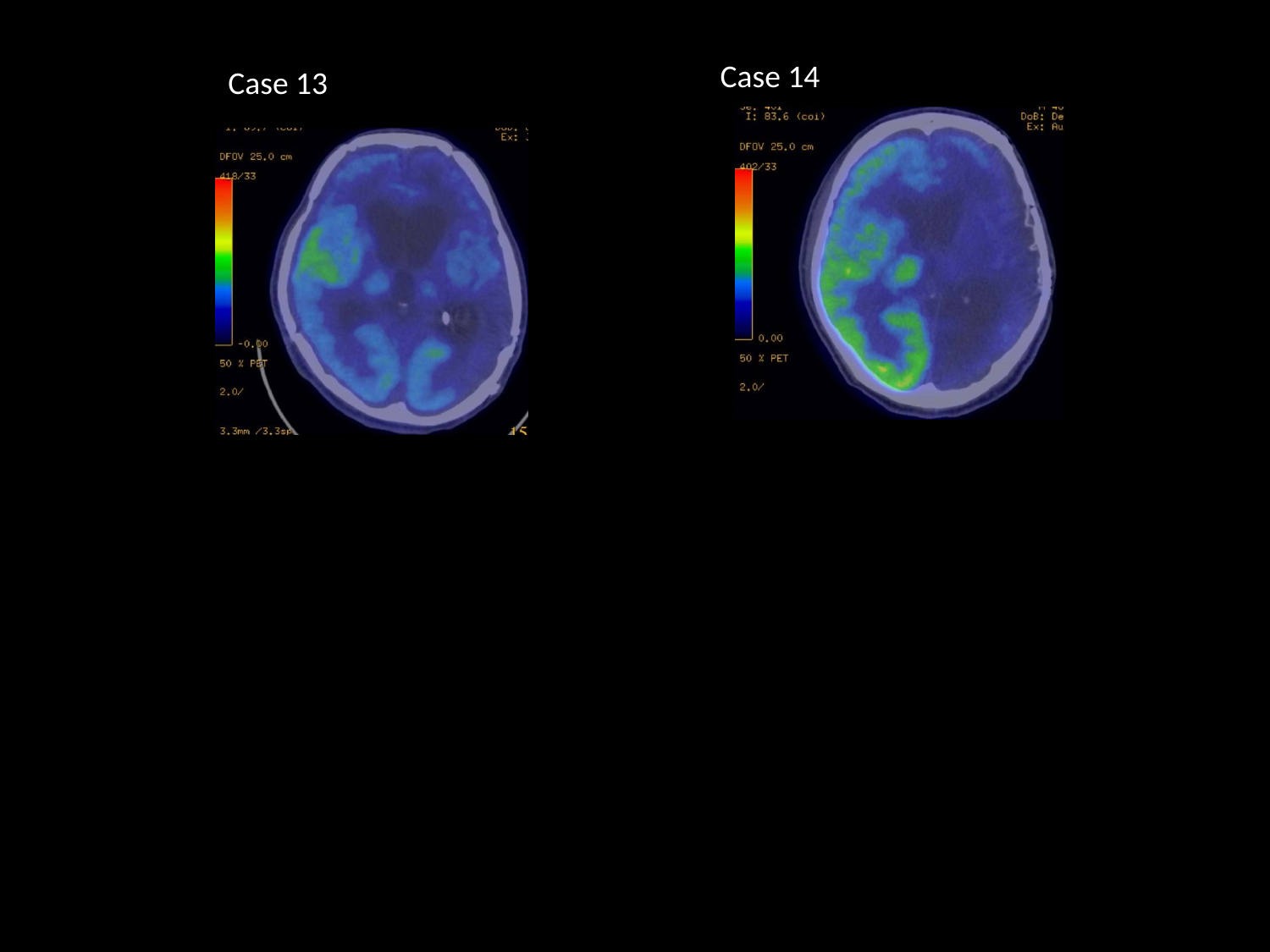

Case 14
Case 13

Supplement: Supplementary file 4 — Figure S2. All available 18F-fluorodeoxyglucose positron emission tomography/computed tomography images for the 14 patients who had severe traumatic brain injury with or without a behavioral disorder. (PPTX 885 kb) [file 13030_2018_125_MOESM4_ESM.pptx]
