# Supplementary material for: Association between uncooperativeness and the glucose metabolism of patients with chronic behavioral disorders after severe traumatic brain injury: a cross-sectional retrospective study
Source: Biopsychosoc Med. 2018 Apr 23;12:6. doi: 10.1186/s13030-018-0125-0 (PMC5914015; doi:10.1186/s13030-018-0125-0)
Supplement: Supplementary file 2 — Table S1. Comparison between the patients who were evaluated for psychiatric assessment (the evaluable group) and those who could not be assessed (the unevaluable group). (DOCX 18 kb) [file 13030_2018_125_MOESM2_ESM.docx]

Supplementary table 1

| Demographic and  clinical data | Total (n = 70) | Evaluable  group (n = 26) | Unevaluable  group (n = 44) | Statistical test.  P value |
| --- | --- | --- | --- | --- |
| Age (years), mean (SD) | 46.0 (18.6) | 47.9 (16.7) | 44.8 (19.7) | T = -0.66, P = 0.25 |
| Male, n (%) | 47 (67.1) | 20 (76.9) | 27 (61.4) | χ^2^ =1.79, P = 0.20 |
| CRS-R score |  |  |  |  |
| Auditory function scale (SD) | 2.0 (1.4) | 3.4 (0.9) | 1.2 (0.9) | P< 0.0001 |
| Visual function scale (SD) | 2.9 (1.9) | 4.4 (1.4) | 2.0 (1.7) | P< 0.0001 |
| Motor scale (SD) | 3.3 (2.2) | 5.5 (0.8) | 2.1 (1.7) | P< 0.0001 |
| Oromotor/verbal scale (SD) | 1.2 (1.1) | 2.1(0.9) | 0.7 (0.9) | P< 0.0001 |
| Communication scale (SD) | 0.6 (0.7) | 1.3 (0.6) | 0.2 (0.4) | P< 0.0001 |
| Arousal scale (SD) | 2.0 (1.1) | 3.0 (0) | 1.4 (1.1) | P< 0.0001 |
| The sum of score of CRS-R (SD) | 12.1 (7.5) | 19.7 (2.9) | 7.6 (5.6) | P< 0.0001 |

Comparison of evaluable group, who were evaluable for psychiatric assessment, and unevaluable group, who were unevaluable for psychiatric assessment.

**P* < 0.05; *CRS-R*, Coma Recovery Scale-Revised; *SD*, standard deviation
